# Factor structure of the brief psychiatric rating scale-expanded among outpatients with psychotic disorders in five Southeast European countries: evidence for five factors

**DOI:** 10.3389/fpsyt.2023.1207577

**Published:** 2023-10-25

**Authors:** Stojan Bajraktarov, Biljana Blazhevska Stoilkovska, Manuela Russo, Selman Repišti, Nadja P. Maric, Alma Dzubur Kulenovic, Aliriza Arënliu, Lidija Injac Stevovic, Ljubisha Novotni, Emina Ribic, Jon Konjufca, Ivan Ristic, Antoni Novotni, Nikolina Jovanovic

**Affiliations:** ^1^University Clinic of Psychiatry, Ss. Cyril in Methodius University in Skopje, Skopje, North Macedonia; ^2^Department of Psychology, Faculty of Philosophy, Ss. Cyril in Methodius University in Skopje, Skopje, North Macedonia; ^3^Unit for Social and Community Psychiatry, WHO Collaborating Centre for Mental Health Services Development, Bart's and The London School of Medicine and Dentistry, Queen Mary University of London, London, United Kingdom; ^4^Faculty of Applied Sciences, University of Donja Gorica, Podgorica, Montenegro; ^5^Faculty of Medicine, Institute of Mental Health, University of Belgrade, Belgrade, Serbia; ^6^Clinical Center University of Sarajevo, Sarajevo, Bosnia and Herzegovina; ^7^Department of Psychology, Faculty of Philosophy, University of Prishtina, Prishtina, Kosovo; ^8^Psychiatric Clinic, Clinical Centre of Montenegro, Faculty of Medicine, University of Montenegro, Podgorica, Montenegro; ^9^Wolfson Institute of Population Health, Queen Mary University of London, London, United Kingdom

**Keywords:** BPRS-E scale, factor structure, principal axis factoring, confirmatory factor analysis, outpatients with psychotic disorders, cross-national study

## Abstract

The Brief Psychiatric Rating Scale (BPRS) is a useful tool for measuring the severity of psychopathological symptoms among patients with psychosis. Many studies, predominantly in Western countries, have investigated its factor structure. This study has the following aims: (a) to further explore the factor structure of the BPRS-Expanded version (BPRS-E, 24 items) among outpatients with psychotic disorders in Southeast European countries; (b) to confirm the identified model; and (c) to investigate the goodness-of-fit of the three competing BPRS-E factor models derived from previous studies. The exploratory factor analysis (EFA) produced a solution with 21 items grouped into five factors, thus supporting the existence of a fifth factor, i.e., Disorganization. A follow-up confirmatory factor analysis (CFA) revealed a 19-item model (with two items removed) that fit the data well. In addition, the stability of two out of three competing factor models was confirmed. Finally, the BPRS-E model with 5 factors developed in this cross-national study was found to include a greater number of items compared to competing models.

## Introduction

The Brief Psychiatric Rating Scale (BPRS) was developed to assess the severity of psychopathology symptoms among people diagnosed with psychosis. Its administration does not take a long time, which makes it appropriate for use in clinical settings in situations when efficacy and speed are needed (1).

The original version of this measure consisted of 16 items indicating symptoms scored on a 7-point scale ranging from 1 (not present) to 7 (severe) (1). Clinicians can easily perform such a rating in 2–3 min after the completion of the 20 min structured interview with the patient. Some items are rated based on the clinician's or interviewer's observation of the patient's behavior, whereas the remaining symptoms are assessed using the information obtained during the interview. In subsequent versions of BPRS, Overall and Gorham (2) included two additional items (BPRS-18 version), while Ventura et al. (3) expanded the scale with six new items/symptoms (BPRS-24 or BPRS-E).

Empirical evidence on the factor structure of BPRS-expanded (BPRS-E) showed 4-, 5-, and 6-factor solutions for this measure. Most of the studies revealed that the symptoms in BPRS-E tend to cluster in four dimensions and found that this factor structure is the most stable and adequately representing groups of symptoms in psychosis. In this context, Velligan et al. (4), using the principal axis factoring method (PAF) with Varimax and Promax rotation, identified four factors of BPRS-E among outpatients with major depression, schizophrenia, and bipolar disorder, i.e., anxiety or depression, psychosis, negative symptoms, and activation. The authors concluded that BPRS-E provides a more comprehensive assessment compared to the initial version of BPRS and should be used with people diagnosed with mental health disorders. Similarly, Kopelowicz et al.'s (5) study found that the same 4-factor solution of BPRS-E [positive symptoms, negative symptoms, anxiety/depression, activation/mania extracted with principal component analysis (PCA), and Varimax rotation] was stable across the course of schizophrenia. Furthermore, research conducted on a sample of individuals with psychosis (non-affective) from 11 European countries (6) used a 6-factor structure of BPRS-E with PCA with Varimax rotation applied during their admission, i.e., mania, negative symptoms, positive symptoms, depression, agitation, and anxiety. In another cross-national study involving outpatients with schizophrenia from five Western European countries, Rugerri et al. (7) identified a 4-factor solution of BPRS-E using PCA (mania/disorganization, positive symptoms, negative symptoms, depression/anxiety) with mania and disorganization symptoms loaded as one factor. Previously, Ventura et al. (8) used PCA with Varimax rotation and identified a 5-factor solution among a relatively small sample consisting of patients with recent-onset schizophrenia and schizoaffective and bipolar disorders; however, they concluded that the fifth factor was not interpretable. In their model, mania and disorganization symptoms were clustered together as the same factor. Two additional studies revealed five underlying factors for the BPRS-E symptoms among general psychiatric inpatients (9, 10). In general, when comparing these results, it is evident that identified models with the same number of factors showed slightly different content. However, core items were typically loaded on the same factors across all models.

Despite these insights, it was suggested that a 4-factor solution is better than a 5-factor structure, even though both are very similar (9, 11). Following this, a meta-analysis of the BPRS-18 factor structure (12) showed that the model with 4 factors represented the core BPRS structure, but it also implied a 5-factor solution that included affect (anxiety, guilt, depression, and somatic concern); positive symptoms (unusual thought content, conceptual disorganization, hallucinations, and grandiosity); negative symptoms (blunted affect, emotional withdrawal, and motor retardation); resistance (hostility, uncooperativeness, and suspiciousness); and activation (excitement, tension, mannerisms-and posturing). Based on a more recent meta-analysis of the BPRS-E structure, Dazzi et al. (13) concluded that the 4-factor model of affect (anxiety, guilt, depression, and suicidality), positive symptoms (hallucinations, unusual thought content, suspiciousness, and grandiosity), negative symptoms (blunted affect, emotional withdrawal, and motor retardation), and activation (excitement, motor hyperactivity, elevated mood, and distractibility) was statistically supported and relatively invariant. Furthermore, the authors recommended adding a fifth factor, the factor of Disorganization (conceptual disorganization, disorientation, self-neglect, and mannerism-posturing), to the model.

The BPRS-E factor structure, derived in the aforementioned meta-analysis, along with models extracted in other studies were tested on a large sample of hospitalized patients. These patients were diagnosed with schizophrenia, major depressive disorder, bipolar disorder, manic episodes, followed by addiction-related disorders, adjustment disorders, dementia, and personality-impulsive disorders (14). The authors reported that the meta-analytic 4-factor model with 12 core BPRS items had an excellent fit, but additionally, a model consisting of 5-factors (including Disorganization) with 15 core BPRS-E symptoms also showed an acceptable fit. Studies conducted among outpatients with unipolar depression (15) and patients with manic episodes (16) revealed a 6-factor and 4-factor solutions of BPRS-E, respectively, with factors specific to the disorder or illness.

Differences in the number of factors in BPRS-E might be linked to differences in the sample size across studies, the type of disorder, and participants' status as inpatients and outpatients, and, possibly, different factor extraction and factor rotation methods applied while examining its latent structure. In addition, all findings come from an American and/or Western European context; thus, further exploration of the BPRS-E structure in other and different contexts can reveal new empirical evidence. Even more, recent studies (13, 14) suggest that additional evidence on the Disorganization factor and the 5-factor model is necessary.

To the best of our knowledge, the factor structure of BPRS-E has not been investigated among outpatients with schizophrenia spectrum disorders and bipolar disorder (F20-29 and F-31) who are in a remission phase, particularly in low- and middle-income Southeast European countries. Moreover, previous findings on the factor model of the BPRS-E psychopathology symptoms have not been consistent.

Therefore, this study aimed to explore the factor structure of BPRS-E on a sample of outpatients with psychotic disorders in the following five Southeast European countries: Bosnia and Herzegovina, Montenegro, Kosovo (UN resolution), North Macedonia, and Serbia. More precisely, its first aim was to examine the underlying groups of psychopathology symptoms as measured by BPRS-E using exploratory factor analysis. The second aim of the study was to further investigate the goodness-of-fit of the identified factor model, applying confirmatory factor analysis, as well as to compare it with three competing, previously derived factor models of BPRS-E, i.e., the 4-factor model with 12 core items, the 4-factor model with 15 items, and the 5-factor model with 15 items [see (13, 14)].

## Materials and methods

### Sample and procedure

The participants in this study were 466 outpatients diagnosed with psychotic disorders from the following five countries: Bosnia and Herzegovina, Montenegro, Kosovo (UN resolution), North Macedonia, and Serbia. The sample was part of the IMPULSE project [see (17)]. The following criteria were considered for the inclusion of patients in the study: primary diagnosis of psychosis or a related disorder, i.e., F20–29 and F31 [ICD-10; (18)], age above 18 years, currently attending regular medical treatment or examinations in the outpatient clinic, a history of at least one psychiatric hospital admission in their lifetime, and the capacity and will to provide informed consent. Patients diagnosed with organic brain disorders and severe cognitive deficits were excluded due to their inability to provide informed consent and reliable information to study instruments. Participants' mean age was 42.64 years (SD = 11.27). Their sociodemographic characteristics and diagnosis are presented in Table 1.

**Table 1:** Sociodemographic characteristics and diagnosis of the study participants (*N* = 466).

	**Frequency**	**Percent**
**Site/country**		
Bosnia and Herzegovina	80	17.2
Macedonia	82	17.6
Kosovo	102	21.9
Montenegro	122	26.2
Serbia	80	17.2
**Sex**		
Female	212	45.5
Male	254	54.5
**Marital status**		
Single–not in a relationship	253	54.3
Married/co-habiting/civil partnership/any partnership	124	26.6
Separated/divorced	75	16.1
Widow/widower	14	3.0
**Level of education**		
Less than elementary school	9	1.9
Elementary school graduate	77	16.5
High school graduate	283	60.7
University or college graduate	85	18.2
Postgraduate or professional qualification	8	1.7
Other	4	0.9
**Diagnosis**		
F20-29	397	85.19
F31	69	14.81

The study was approved by the ethical committees in all participating countries. Bosnia and Herzegovina—approval No. 03-02-4216 and 02.8-408/19 (Klinicki Centar Univerziteta u Sarajevu, Eticki Komitet 03-02-4216, Eticki Komitet JU Psihijatrijska bolnica Kantona Sarajevo i JU Zavod za bolesti ovisnosti Kantona Sarajevo, 02.8-408/19); Serbia—approval No. 2650/XII-20 and 01-36/1 (Eticka komisija Medicinskog fakulteta u Beogradu 2650/XII-20 and Eticka Komisija Specijalne bolnice “Dr Slavoljub Bakalovic” Vrsac, 01-36/1); Kosovo—approval No. 209-85 (Hospital and University Clinical Service of Kosovo, Ethics Committee 2019-85); Republic of North Macedonia—approval No. 03-24219 (Eticka Komisija za istrazuvanje na luge, Medicinski Fakultet pri UKIM vo Skopje, 03-24219); and Montenegro—approval No. 03/01–29304/1 and 01-47 (Javna zdravstvena ustanova Klinicki centar Crne Gore, Eticki Komitet 03/01–29304/1, ZU Specijalna bolnica za psihijatriju “Dobrota” Kotor, Eti cki Komitet, Eticki Komitet JZU Dom ydravlja “DR Nika Labovic” Berane 01-47). All participants provided written informed consent prior to the study.

The data were collected from January to April 2019 in hospital centers where participants received outpatient mental healthcare services. Some patients were invited to come in at other times suitable for them during the working days. The interview and assessment lasted for 15–20 min. All researchers (psychiatrists and psychologists) were trained in administering BPRS-E (ICC registered after the training was above 0.80).

### Measure

The Brief Psychiatry Rating Scale-Expanded (BPRS-E; 3) with 24 items was applied to assess psychopathological symptoms in study participants. Following the interview questions and rating guidelines provided by the authors, all items were rated on a 7-point Likert scale ranging from 1 (not present) to 7 (extremely severe). Higher scores indicated more severe symptomatology.

### Data analysis

The study sample was randomly divided into two groups of participants. First, an exploratory factor analysis (principal axis factoring with Promax rotation) was performed on a calibration subsample (*n* = 226). The principal axis factoring was applied because it does not assume a normal distribution of the study variables (19), while oblique rotation was chosen to provide a more easily interpretable solution when correlation among factors is expected (20).

Kaiser-Meyer-Olkin's measure of adequacy of 0.765 and statistically significant Bartlett's test of sphericity (χ^2^ = 1867.32, df = 276, *p* < 0.001) indicated that the data were suitable for factor analysis. Factor loadings >0.32 (21) were considered, while an eigenvalue >1 was used as a criterion for the number of factors extracted.

In the follow-up analysis conducted on the second validation subsample (*n* = 240), the BPRS-E factor structure identified in this study was tested using confirmatory factor analysis with the weighted least squares mean and variance adjusted (WLSMV) method for the estimation of parameters. This estimator was reported to be a suitable alternative to the well-known maximum likelihood (ML) method when there is a severe deviation from the normal distribution of the examined variables (22). Considering Kline's (23) recommendation, the following indices were used for model fit evaluation: χ^2^ test statistic, comparative fit index (CFI), root mean square error of approximation (RMSEA), and standardized root mean square residual (SRMR). According to Hu and Bentler (24), values of CFI ≥ 0.95, RMSEA ≤ 0.06, and SRMR ≤ 0.08 were considered as the criteria for a good model fit. However, values below 0.90 for CFI and above 0.10 for RMSEA and SRMR indicated that the model fit was not acceptable (25). The comparison of the competing models was based on the change in CFI value, i.e., ΔCFI ≤ 0.01 (26). This procedure was employed to test three additional and competing models of BPRS-E, i.e., the 4-factor model with 12 core items, the 4-factor model with 15 items, and the 5-factor model with 15 items.

Descriptive analysis of the items/variables, reliability analysis, and EFA were performed with SPSS v.28. CFA was conducted using the lavaan package (27) in the R environment (28).

## Results

Descriptive statistics (mean, standard deviation, and minimum and maximum scores) of all variables, items, and symptoms in BPRS-E are presented in Table 2. Data on median, skewness, and kurtosis are given in Supplementary Table 1.

**Table 2:** Descriptive statistics of the BPRS-E items for two subsamples—calibration (*n* = 226) and validation (*n* = 240).

**Symptoms/Items**	**M**		**SD**		**Min/Max**	
	***n*** = **226**	***n*** = **240**	***n*** = **226**	***n*** = **240**	***n*** = **226**	***n*** = **240**
Somatic concern	2.46	2.53	1.58	1.60	1/7	1/7
Anxiety	2.77	2.83	1.66	1.70	1/7	1/7
Depression	2.65	2.66	1.56	1.62	1/7	1/7
Suicidality	1.43	1.42	0.90	0.94	1/6	1/7
Guilt	1.97	0.97	1.21	1.20	1/5	1/5
Hostility	1.59	1.60	0.96	0.98	1/5	1/5
Elevated mood	1.51	1.47	0.96	0.95	1/7	1/7
Grandiosity	1.31	1.34	0.93	1.04	1/7	1/7
Suspiciousness	2.15	1.90	1.43	1.26	1/7	1/6
Hallucinations	1.73	1.60	1.40	1.25	1/7	1/7
Unusual thought content	1.72	1.54	1.30	1.16	1/6	1/7
Bizarre behavior	1.32	1.28	0.81	0.84	1/5	1/7
Self-neglect	1.63	1.59	0.95	0.99	1/6	1/5
Disorientation	1.39	1.35	0.85	0.76	1/6	1/6
Conceptual disorganization	1.51	1.56	0.96	1.03	1/6	1/6
Blunted affect	2.68	2.39	1.55	1.42	1/6	1/7
Emotional withdrawal	2.07	1.90	1.24	1.16	1/7	1/6
Motor retardation	2.04	1.93	1.29	1.13	1/6	1/6
Tension	2.02	1.87	1.16	1.06	1/6	1/6
Uncooperativeness	1.25	1.23	0.64	0.65	1/5	1/5
Excitement	1.62	1.53	1.02	0.86	1/7	1/5
Distractibility	1.84	1.93	1.03	1.13	1/6	1/6
Motor hyperactivity	1.42	1.42	0.83	0.83	1/5	1/6
Mannerisms and posturing	1.37	1.23	0.79	0.59	1/5	1/4
Overall BPRS-E	1.81	1.75	0.50	0.52	1/3.17	1/3.42

Six factors with an eigenvalue >1 were extracted, accounting for 47.95% of the variance of the BPRS-E symptomatology. The proportion of explained variance by each factor is given in Table 3.

**Table 3:** Exploratory factor analysis: factor loadings, communalities, explained variance, and correlation among factors (*n* = 226).

	**Factors**						
**Items**	**F 1**	**F 2**	**F 3**	**F 4**	**F 5**	**F 6**	**Communalities**
Depression	0.816						0.688
Anxiety	0.690						0.541
Suicidality	0.627						0.383
Guilt	0.543						0.296
Somatic concern	0.429						0.310
Blunted affect		0.875					0.767
Emotional withdrawal		0.788					0.649
Motor retardation		0.699					0.545
Mannerisms and posturing		0.448					0.437
Hallucinations			0.744				0.467
Unusual thought content			0.741				0.575
Bizarre behavior			0.662				0.546
Suspiciousness			0.483				0.463
Motor hyperactivity				0.771			0.565
Excitement				0.744			0.654
Elevated mood				0.529		0.328	0.253
Tension				0.485			0.407
Conceptual disorganization					0.860		0.660
Distractibility					0.498		0.570
Disorientation					0.367	0.495	0.397
Grandiosity			0.432			0.462	0.419
Explained variance	17.88	10.45	8.51	5.91	2.76	2.46	
F1		0.13^**^	0.34^***^	0.19^***^	0.20^***^		
F2			0.14^**^	−0.13^*^	0.30^***^		
F3				0.16^**^	0.26^***^		
F4					0.15^**^		
Cronbach's alpha	0.75	0.77	0.74	0.65	0.65		

F1, Affect; F2, Negative symptoms; F3, Positive symptoms; F4, Activation; F5, Disorganization. ^*^p < 0.05, ^**^p < 0.01, ^***^p < 0.001.

The first factor, named anxiety/depression (affect), consisted of somatic concern, anxiety, depression, suicidality, and guilt. Flat affect, emotional withdrawal, motor retardation, and mannerism were loaded on the second factor called negative symptoms. The third factor, called positive symptoms, consisted of hallucinations, unusual thought content, bizarre behavior, suspicion, and grandiosity. The fourth factor, called activation, comprised motor hyperactivity, excitement, tension, and elevated mood items/symptoms. Conceptual disorganization, distractibility, and disorientation were loaded on the fifth factor named Disorganization. The sixth factor consisted of three items, namely, elevated mood, disorganization, and grandiosity, that were all cross-loaded on other factors (i.e., on the fourth, fifth, and third factors, respectively). As a result, these three items were retained in those factors. In addition, the scree plot indicated a solution with five factors as well. Hostility, self-neglect, and uncooperativeness did not load to any extracted factor (factor loadings < 0.32). These items were removed from the obtained model and further analysis.

Cronbach's alpha reliability coefficients ranged from 0.65 to 0.77 (see Table 3). As seen in Table 3, an association of negative symptoms with anxiety and depression (affect) group of symptoms, positive symptoms, and activation was statistically significant but weak (*r* = 0.13, *p* < 0.01; *r* = 0.14, *p* < 0.01, and *r* = −0.13, *p* < 0.05, respectively). Positive symptomatology and negative symptomatology factors were significantly and moderately related to the disorganization group of symptoms (*r* = 0.26, *p* < 0.001, and *r* = 0.30, *p* < 0.001, respectively). This implied that the factors of Disorganization and cognitive impairment symptoms go along with other identified groups of symptoms characteristic of psychotic disorders. The relationship of disorganization to anxiety and depression and activation was found to be weaker (*r* = 0.20, *p* < 0.01 and *r* = 0.15, *p* < 0.01, respectively).

A follow-up analysis using CFA revealed mixed results regarding model fit, as shown in Table 4. More precisely, CFI was under the recommended value, while RMSEA and SRMR implied an acceptable data fit. In addition, the factor loading of grandiosity symptom on positive symptoms factor was very low and non-significant. Grandiosity, along with the tension symptom, had correlated residuals with other items/symptoms. When these two items were deleted, the CFI value increased (ΔCFI = 0.13), and RMSEA and SRMR indices slightly decreased, demonstrating evidently a better fit of the tested factor model (Table 4). Further, the results showed that the primary factor loadings of elevated mood, mannerism, and bizarre behavior items were 0.24, 0.28, and 0.30, respectively (Table 5, 19-item 5-factor model of this study). Given that these factor loadings were statistically significant (*p* < 0.01), the items were retained in the factor model.

**Table 4:** Confirmatory factor analysis: goodness-of-fit indices of the tested BPRS-E factor models (*n* = 240).

**Model**	**χ^2^**	**CFI**	**ΔCFI**	**RMSEA [90% CI]**	**SRMR**
21-item, 5-factor (this study model)	266.53	0.78		0.05 [0.04, 0.06]	0.09
19-item, 5-factor (this study model)	189.95	0.91	0.13	0.04 [0.02, 0.05]	0.07
15 core items, 4-factor (original model)	197.08	0.70		0.08 [0.06, 0.09]	0.10
12 core items, 4-factor (best fitting model)	63.39	0.94	0.16	0.04 [0.00, 0.06]	0.06
15-item, 5-factor (alternative model)	109.95	0.91	0.13	0.04 [0.02, 0.06]	0.06

χ^2^, chi square test statistic; CFI, comparative fit index; ΔCFI, changes in the comparative fit index; RMSEA, root mean square error of approximation; SRMR, standardized root mean square residual.

**Table 5:** Factor loadings for the models tested in the confirmatory factor analysis (*n* = 240).

	**21-item, 5-factor (this study model)**	**19-item, 5-factor (this study model)**	**15 core items, 4-factor (original model)**	**12 core items, 4-factor (best fitting model)**	**15-item, 5-factor (alternative model)**
**Affect**					
Depression	0.804	0.801	0.818	0.767	0.767
Anxiety	0.772	0.772	0.778		
Suicidality	0.612	0.636	0.663	0.648	0.645
Guilt	0.608	0.599	0.610	0.630	0.632
Somatic concern	0.666	0.665			
**Negative symptoms**					
Blunted affect	0.786	0.790	0.812	0.821	0.816
Emotional withdrawal	0.823	0.812	0.721	0.705	0.752
Motor retardation	0.865	0.876	0.926	0.929	0.893
Mannerisms and posturing	0.293	0.284			
**Positive symptoms**					
Suspiciousness	0.843	0.817	0.849	0.863	0.823
Hallucinations	0.481	0.503	0.452	0.444	0.475
Unusual thought content	0.470	0.461	0.411	0.441	0.443
Bizarre behavior	0.314	0.304			
Grandiosity	0.015		-0.031		
**Activation**					
Motor hyperactivity	0.742	0.778	0.820	0.794	0.776
Excitement	0.638	0.610	0.665	0.654	0.624
Elevated mood	0.144	0.240	0.200	0.193	0.231
Tension	0.627				
**Disorganization**					
Conceptual disorganization	0.686	0.692			0.579
Distractibility	0.839	0.818	0.211 (in Factor 4)		
Disorientation	0.573	0.593			0.542
Self-neglect					0.739

The analysis of the competing BPRS-E factor models revealed that the best-fitted model in this study was the 4-factor solution with 12 core items. While the model with five factors and 15 items or symptoms showed an acceptable fit, the model with 15 symptoms grouped into four factors (Table 4) did not. However, the difference between the 19-item model obtained in this study and the two competing models was trivial, particularly with the 15-item 5-factor alternative model (Table 4). Factors and factor loadings for all tested models are presented in Table 5.

## Discussion

This cross-national study provided evidence on the factor structure of the BPRS-E among outpatients with psychosis in Southeast European countries, thus contributing to the existing empirical findings that generally come from Western countries. To the best of our knowledge, the previously mentioned study on BPRS-E structure (6) included participants with acute psychopathology symptoms during their hospital admission from two Southeast European countries, both of which are EU member states, along with participants from nine West and East European countries. Additionally, participants from nine West and East European countries with acute psychopathology symptoms were included, and these data were collected during their hospital admission.

Exploratory factor analysis revealed a factor solution with 21 items and similar clustering of core psychopathology symptoms, as measured by BPRS-E, found in previous research [e.g., (9)]. However, there were differences in the distribution of some symptoms across the extracted factors that might be explained by different factor extraction and factor rotation methods used, alongside variations associated with sample size and participants' diagnoses.

It should be particularly emphasized that the CFA findings in this study supported the BPRS-E model, which contains 19 items grouped into five factors, with Disorganization as a fifth factor. Furthermore, its content was similar to that reported in Shafer et al.'s (14) study, clearly referring to cognition impairment (thinking, speech, attention). For instance, this factor consisted of conceptual disorganization, disorientation, and distraction symptoms. The symptoms of conceptual disorganization, disorientation, and distraction were all part of the Disorganization factor, in the study by Horton and Silverstein (29), but along with positive symptoms. However, the core symptoms of conceptual disorganization and disorientation were both found to load on this factor in other studies as well [e.g., (5, 13, 30)].

Consistent with other findings [e.g., (7, 8, 10)], in this study, the symptom of mannerism-posturing loaded on the negative symptoms factor along with blunted affect, emotional withdrawal, and motor retardation. However, three out of four symptoms that were excluded from the BPRS-E model, i.e., uncooperativeness, hostility, and tension, included resistance as a sixth factor in the factor structure obtained among hospitalized patients in the study using EFA (14). These items were also found to load on the resistance factor in other studies [e.g., (31)]. Such findings might imply that these symptoms emerged as a particular group in hospitalized or more severely ill patients but did not contribute to the symptomatology, as measured by BPRS-E, when it comes to mildly ill patients or outpatients in the remission stage of illness.

It should be noted that the model identified in this study showed an acceptable fit, very similar to that of the competing models (12-core item 4-factor model and 15-item 5-factor alternative model). More precisely, the factor structure of the BPRS-E psychopathology symptoms identified in this study showed identical clustering of the core symptoms as in the competing 15-item 5-factor alternative model. However, it is more inclusive since it contains a greater number of items−19 in total. In addition, this model confirmed the stability of affect (anxiety/depression) and negative symptomatology factors. Furthermore, the findings implied that the main symptoms of psychotic disorders with all factors show acceptable internal consistency.

This study has several strengths and limitations. The data were collected by trained researchers whose ratings demonstrated high inter-rater reliability. The research was carried out in five low- and middle-income countries in Southeast Europe with similar cultural, socioeconomic, and healthcare systems. The sample was relatively large, consisting of outpatients with psychotic spectrum disorders in remission and stable stage of the illness. In that context, this study provides an important contribution to the existing findings on the BPRS-E factor structure obtained in Western countries. It should be noted that most of the previous studies used principal component analysis to investigate the factor structure of BPRS-E. In this study, factor analysis was applied as a more suitable method when underlying factors needed to be identified. However, some variations in the BPRS-E factor structure are possible, but considering the aforementioned similarities, it could be assumed that the identified structure is applicable across five countries. Data on illness duration, number of past hospitalizations, and pharmacotherapy were not taken into account, which could be considered as a limitation of the obtained results on psychopathology symptoms measured with BPRS-E. In addition, the research was focused on construct validity; therefore, future studies aiming to investigate the predictive and convergent validity of BPRS-E are needed.

## Conclusion

Exploratory factor analysis of BPRS-E yielded a solution with six factors, among which five were clearly defined. Extracted factors consisted of 21 items/symptoms, generally in accordance with the previously produced factor models. A follow-up confirmatory factor analysis revealed a 19-item solution (i.e., a model with two additional items removed) that fitted the data well. The results implied that the two previously identified BPRS-E factor models tested in this study were stable: the 12-core item 4-factor model and the 15-item 5-factor alternative model.

The findings of this study clearly indicate the stability of four BPRS-E factors, i.e., affect, negative symptoms, positive symptoms, and activation. They further pointed out the existence of a fifth factor—the disorganization group of symptoms in a sample of outpatients with psychosis in the Southeast European context. In addition, this model was found to include more items and symptoms compared to competing models. The results revealed acceptable to good reliability of all five factors/groups of symptoms.

It could be concluded that the scale may be used for research purposes considering its factor structure and internal consistency. Furthermore, this measure may be used in a clinical setting for assessing the severity of psychopathology symptoms among outpatients with psychotic disorders in the Balkans, along with other psychopathology assessment methods. The results could be useful to researchers and clinicians, particularly in the Balkans, representing additional empirical evidence on the factor structure of BPRS-E that supported the existence of the fifth factor of Disorganization.

## Data availability statement

The datasets presented in this article are not readily available for confidentiality reasons. Requests to access the datasets should be directed to NJ.

## Ethics statement

The study was approved by the Ethical Committees in all participating countries: Bosnia and Herzegovina–approval No. 03-02-4216 and 02.8-408/19 (Klinicki Centar Univerziteta u Sarajevu –Eticki Komitet 03-02-4216, Eticki komitet JU Psihijatrijska bolnica Kantona Sarajevo i JU Zavod za bolesti ovisnosti Kantona Sarajevo, 02.8-408/19), Serbia–approval No. 2650/XII-20 and 01-36/1 (Eticka komisija Medicinskog fakulteta u Beogradu 2650/XII-20 and Eticka komisija Specijalne bolnice Dr. Slavoljub Bakalovic Vrsac, 01-36/1), Kosovo-approval No. 209-85 (Hospital and University Clinical Service of Kosovo–Ethics Committee 2019-85), Republic of North Macedonia–approval No. 03-24219 (Eticka komisija za istrazuvanje na luge, Medicinski Fakultet pri UKIM vo Skopje, 03-24219), and Montenegro-approval No. 03/01–29304/1 and 01-47 (Javna zdravstvena ustanova Klinicki centar Crne Gore–Eticki komitet 03/01–29304/1, ZU Specijalna bolnica za psihijatriju Dobrota Kotor–Eticki komitet, Eticki komitet JZU Dom ydravlja Dr. Nika Labovic Berane 01-47). All participants provided written informed consent prior to the study. The studies were conducted in accordance with the local legislation and institutional requirements. The participants provided their written informed consent to participate in this study.

## Author contributions

SB: writing—review and editing, conceptualization, resources (study materials and participants), leadership, and coordination responsibility for research activity and execution. BB: study conceptualization, methodology, validation, statistical analyses, data collection and curation, and writing—original draft. MR: conceptualization, validation, data curation, writing—review and editing, and coordination of research activity planning. SR: coneptualization, data collection and curation, and review and editing. NM: review and editing, resources (study materials and participants), leadership, and coordination responsibility for research activity and execution. AD, AA, and LS: resources (study materials and participants), leadership, and coordination responsibility for research activity and execution. LN, ER, JK, and IR: data collection and data curation. AN: writing—review and editing, supervision, project administration, resources, and investigation. NJ: review and editing, management and coordination responsibility for research activity planning and execution, and funding acquisition. All authors contributed to the article and approved the submitted version.
