# Supplementary material for: Factor structure of the brief psychiatric rating scale-expanded among outpatients with psychotic disorders in five Southeast European countries: evidence for five factors
Source: Front Psychiatry. 2023 Oct 25;14:1207577. doi: 10.3389/fpsyt.2023.1207577 (PMC10634518; doi:10.3389/fpsyt.2023.1207577)
Supplement: Supplementary file 1 [file Table_1.pdf]

Table S1
Descriptive statistics of the BPRS-E items for two subsamples-calibration (n=226) and validation (n=240)

Symptoms	Mdn		Sk		Kk	
	n=226	n=240	n=226	n=240	n=226	n=240
Somatic concern	2.00	2.00	.68	.62	-.73	-.94
Anxiety	3.00	3.00	.68	.65	-.49	-.60
Depression	2.00	2.00	.68	.67	-.52	-.53
Suicidality	1.00	1.00	2.76	2.89	8.55	9.52
Guilt	2.00	2.00	1.10	1.21	.18	.68
Hostility	1.00	1.00	1.66	1.69	1.97	2.02
Elevated mood	1.00	1.00	2.77	2.34	8.66	6.12
Grandiosity	1.00	1.00	3.84	3.54	16.65	12.76
Suspiciousness	2.00	1.00	1.28	1.34	.93	.91
Hallucinations	1.00	1.00	1.85	2.23	2.34	4.21
Unusual thought content	1.00	1.00	1.89	2.58	2.68	6.51
Bizarre behavior	1.00	1.00	2.80	3.77	7.43	15.62
Self-neglect	1.00	1.00	1.63	1.78	2.60	2.57
Disorientation	1.00	1.00	3.04	2.75	10.52	9.14
Conceptual disorganization	1.00	1.00	2.21	2.10	4.28	4.07
Blunted affect	2.00	2.00	.56	.88	-.80	-.07
Emotional withdrawal	2.00	2.00	1.31	1.53	1.47	2.17
Motor retardation	2.00	2.00	1.18	1.21	.56	.91
tension	2.00	2.00	1.19	1.31	.97	1.55
Uncooperativeness	1.00	1.00	3.38	3.42	13.49	12.96
Excitement	1.00	1.00	2.13	1.82	5.34	3.39
Distractibility	2.00	2.00	1.47	1.35	2.61	1.69
Motor hyperactivity	1.00	1.00	2.17	2.36	2.28	6.23
Mannerisms and Posturing	1.00	1.00	2.28	2.74	4.67	7.37
Overall BPRS-E	1.79	1.67	.56	.96	-.20	.70
